# Effect of Heat Treatment on Osteoblast Performance and Bactericidal Behavior of Ti6Al4V(ELI)-3at.%Cu Fabricated by Laser Powder Bed Fusion

**DOI:** 10.3390/jfb14020063

**Published:** 2023-01-23

**Authors:** Anna Martín Vilardell, Vanesa Cantillo Alzamora, Luana Vittoria Bauso, Cristina Madrid, Pavel Krakhmalev, Mihaela Albu, Ina Yadroitsava, Igor Yadroitsev, Natalia Garcia-Giralt

**Affiliations:** 1 Department of Engineering and Physics, Karlstad University, 651 88 Karlstad, Sweden; 2 IMIM (Institut Hospital del Mar d’Investigacions Mèdiques), CIBERFES, ISCIII, Doctor Aiguader 88, 08003 Barcelona, Spain; 3 Department of Clinical and Experimental Medicine, University of Messina, Consolare Valeria 1, 98125 Messina, Italy; 4 Department of Genetics, Microbiology and Statistics, Faculty of Biology, Universitat de Barcelona, Av. Diagonal 643, 08028 Barcelona, Spain; 5 Graz Centre for Electron Microscopy, Steyrergasse 17, 8010 Graz, Austria; 6 Department of Mechanical Engineering and Mechatronics, Central University of Technology, Bloemfontein 9300, South Africa

**Keywords:** laser powder bed fusion, Ti–Cu alloys, heat treatment, microstructure, osteoblast activity, bactericidal effect

## Abstract

Cu addition to alloys for biomedical applications has been of great interest to reduce bacterial growth. In situ-alloyed Ti6Al4V(ELI)-3at.%Cu was successfully manufactured by laser powder bed fusion (L-PBF). Even so, post-heat treatments are required to avoid distortions and/or achieve required/desired mechanical and fatigue properties. The present study is focused on the investigation of microstructural changes in L-PBF Ti6Al4V(ELI)-3at.%Cu after stress relieving and annealing treatments, as well as their influence on osteoblast and bactericidal behavior. After the stress relieving treatment, a homogenously distributed β phase and CuTi_2_ intermetallic precipitates were observed over the αʹ matrix. The annealing treatment led to the increase in amount and size of both types of precipitates, but also to phase redistribution along α lamellas. Although microstructural changes were not statistically significant, such increase in β and CuTi_2_ content resulted in an increase in osteoblast proliferation after 14 days of cell culture. A significant bactericidal behavior of L-PBF Ti6Al4V(ELI)-3at.%Cu by means of ion release was found after the annealing treatment, provably due to the easier release of Cu ions from β phase. Biofilm formation was inhibited in all on Cu-alloyed specimens with stress relieving but also annealing treatment.

## 1. Introduction

Osteoarthritis (OA) is a degenerative joint disease caused by cartilage damage. The symptoms of osteoarthritis include pain, stiffness, and swelling in the affected joint, which worsen over time [1,2]. The goal of surgery is to remove the injured cartilage and bone and replace it with a prosthesis. After surgery, antibiotics are used as prophylaxis in order to avoid the development of infections [3]. Unfortunately, biofilm formation and the massive use of antibiotics have led to the development of resistant bacterial strains, resulting in a high risk of infection and, as a consequence, the failure of the whole implant [1].

The most common bacteria involved in prosthetic infections are *Staphylococcus aureus* (*S. aureus*) and coagulase-negative staphylococci (CoNS). They account for more than half of prosthetic hip and knee infections, followed by other bacteria such as *Escherichia coli (E. coli)*, *Pseudomonas aeruginosa*, *Proteus mirabilis*, and *Klebsiella pneumoniae*. Such bacteria are capable of forming a biofilm that is resistant to treatments that allow bacterial growth [4]. A biofilm is defined as a microbially derived sessile community characterized by cells that are irreversibly attached to a substratum or interface or to each other. They are embedded in a matrix of extracellular polymeric substances that they have produced and exhibit an altered phenotype with respect to growth rate and gene transcription [5]. Thus, the development of surfaces that prevent the development of infections is required. 

Copper (Cu) is an essential trace element necessary for human development due to its catalyzing metabolic processes (e.g., bone formation and angiogenesis), but it is also known for its antibacterial properties and limited cytotoxicity [6]. In 2008, copper was recognized by the United States Environmental Protection Agency (EPA) as the first metallic antimicrobial agent [7]. It has demonstrated several times its antiviral, antifungal, and antibacterial effects. Because of that, several approaches have been used on titanium–copper-based alloys, as titanium alloys, especially Ti6Al4V, have excellent mechanical properties and good biocompatibility [8]. 

Titanium–copper-based alloys have been successfully manufactured by laser powder bed fusion (L-PBF). This additive manufacturing (AM) technique uses a high-power-density laser that melts and fuses powder into a desired shape. The powder is deposited layer by layer until the final object is built [9,10]. Compared to conventional manufacturing technologies, AM offers higher efficiency, reduced cost of fabrication, and lower energy consumption [11]. 

Generally, titanium–copper-based alloys lead to a microstructure composed of α′ martensite, β phase, and CuTi_2_ intermetallic precipitates [12]. The distribution of Cu within the matrix has a crucial influence/impact on the bactericidal behavior, as Cu ions are hard to release from CuTi_2_ intermetallic because CuTi_2_ has a higher binding force for Cu compared to solid solution Cu atoms [12,13]. The release of Cu ions is considered one of the bactericidal mechanisms, as well as direct contact with the implant’s surface [7]. Because of that, the amount of Cu content should be considered. Liu et al. [14] produced Ti–Cu alloys via hot pressure sintered and reported that the alloy should contain a minimum of 5 wt.% Cu for a strong and stable antibacterial rate of 99.2% against *E. coli* and 99.0% against *S. aureus*. For L-PBF Ti6Al4V–Cu, a strong and stable antibacterial behavior is exhibited against *E. coli* and *S. aureus* at 4 and 6 wt.% Cu, with a rate of 97.40% against *E. coli* and 93.56% against *S. aureus* (for 4 wt.% Cu) and larger than 99% for 6 wt.% Cu [15]. Even so, bactericidal effects have been reported on Ti6Al4V with the addition of 1 at.% Cu [16]. In vivo studies have also confirmed the bactericidal activity of Ti6Al4V–Cu produced by the melting of ingots in a rat model of implant-associated infection [17]. 

After the manufacturing of titanium-based parts, a stress relieving treatment is required to avoid dimensional distortion of the parts, and sometimes, even a heat treatment is required to achieve specific or desired mechanical properties. The microstructure obtained after post-heat treatments should be understood since it leads to changes in mechanical and biological properties. For Ti6Al4V-5 wt.% Cu obtained by an electrode arc-melting furnace, a higher solution temperature could increase the antibacterial ability of the alloy, but at the same time it could change the ductility of the material depending on the amount of α and β phases as well as grain coarsening [18]. Additionally, the presence of the CuTi_2_ intermetallic phase should be considered, as it provides an increase in strength and hardness to the alloy [12,19]. Thus, an appropriate temperature at which both the ductility and the antibacterial performance of the alloy are satisfactory is required. Studies on heat treatments on Ti6A4V–Cu parts obtained by hot forging have been performed [13,18,20], but further studies on L-PBF Ti6A4V–Cu parts should be considered due to the differences in the manufacturing method and, therefore, microstructure. 

In vitro studies show that Ti6Al4V–Cu with up to 7.5 wt.% of Cu does not present cytotoxicity, and Cu contents between 5 and 6 wt.% promote good osteoblast proliferation and differentiation in cast Ti alloys [21]. In previous research [6,12], L-PBF Ti6Al4V(ELI)-3 at.% Cu showed that alloy with 3 at.% (or 4.1 wt.%) Cu addition had a fairly homogeneous microstructure. No cytotoxic effects and good cell attachment were observed for any of the studied conditions for the entire duration of cell culture, indicating the non-cytotoxicity of the L-PBF Ti6Al4V(ELI) with 3 at.% Cu content. The goal of the present research is to understand and correlate the changes in the microstructure of L-PBF Ti6Al4V(ELI)-3 at.% Cu after stress relieving (SR) and annealing (HT) treatments with the bactericidal behavior. For that purpose, microstructural studies were carried out by means of scanning and high-resolution transmission electron microscopies, as well as in vitro studies with Gram-positive (*S. aureus*) and Gram-negative (*E. coli*) bacteria. Additionally, osteoblast viability, proliferation, and mineralization were in vitro tested.

## 2. Materials and Methods

### 2.1. Sample Preparation 

In this study, Ti6Al4V(ELI)-3 at.% Cu alloyed materials were produced in situ by laser powder bed fusion (L-PBF) using an EOSINT M280 system (EOS GmbH, Krailling, Germany). The feedstock powders, manufacturing process parameters, and scanning strategy are described in detail elsewhere [12]. For the in vitro studies, thin vertical rectangular samples of 1 mm thickness were printed. Samples attached to the baseplate were stress-relieved in a vacuum furnace for 3 h at 650 °C with furnace cooling for 10 h. Subsequently, samples were cut off from the baseplate and sliced into 5 × 5 × 1 mm^3^ square samples by wire-cut electrical discharge machining. Half of the specimens were used in SR condition, and the rest were annealed for 2 h at 950 °C in a vacuum furnace with controlled cooling in a furnace for 4 h. The chamber pressure in the vacuum furnace during SR and HT was 10^−7^ bars. The received samples were named Ti6Al4V(ELI)-3Cu (SR) and Ti6Al4V(ELI)-3Cu (HT) correspondingly. Additionally, L-PBF Ti6Al4V(ELI) samples without Cu addition were used as a reference set, after SR [22]. For all the experiments, the surfaces of the samples were ground with 1200 SiC paper. 

### 2.2. Microstructural Characterization

Microstructural and elemental analyses were performed on the cross-section of the specimens by a scanning electron microscope (SEM) JSM-6610A operated at 20 kV and equipped with an energy dispersive spectroscopy detector (EDX). Deeper microstructural investigations were performed by high-resolution scanning transmission electron microscopy (HR-STEM) using a Cs-corrected FEI Titan 3 G2 60–300 STEM operated at 300 keV. The microscope is equipped with a Super X detector (4 Si-drift detectors) for chemical analyses by X-ray spectroscopy. HR-STEM samples were prepared by argon ion milling at cryogenic temperatures. VELOX software was used for elemental mapping, line profile acquisition, and data analysis [23]. Element quantification was performed by using the K-factor method [24]. HR-STEM micrographs were recorded with the annular dark field (ADF) and high angular annular dark field (HAADF) detectors and processed using the Digital Micrograph software. X-ray diffraction (XRD) measurements were conducted using a Cu-Kα radiation source (wavelength λ = 0.15405 nm) operated at 40 kV and 40 mA to identify constituent phases.

### 2.3. Osteoblast Culture

Human primary osteoblasts (hOB) were obtained from the knee trabecular bone after prosthesis replacement following the protocol described by Nacher et al. [25]. Osteoblast samples were obtained from the Parc de Salut Mar Biobank, ERyME (n° 2015/6095/I) and approved by the Parc de Salut Mar Ethics Committee. The study was conducted in accordance with the Helsinki Declaration of 1975. All subjects gave their informed consent for the inclusion of samples in the Biobank. Briefly, the trabecular bone was dissected into small pieces, washed in phosphate-buffered solution (PBS), and placed into a 15 cm diameter Petri dish containing 15 mL of Dulbecco’s Modified Eagle’s Medium (DMEM) supplemented with 10% fetal bovine serum (FBS), penicillin (100 UI/mL), streptomycin (100 UI/mL), ascorbic acid (100 mg/mL) (Invitrogen, Waltham, MA, USA), and fungizone (0.4%) (Gibco, Waltham, MA, USA). Explants were incubated at 37 °C in a humidified atmosphere of 5% CO_2_, changing the medium once a week until cell confluence was achieved. Finally, cells were moved into new 75 cm^2^ flasks until a suitable number was reached. A maximum of 3 subcultures were used in the experiments. For in vitro biomaterial testing, materials were sterilized overnight in ethanol at 70 °C, washed in PBS, and placed on a 48-well polystyrene culture plate (Nunc A/S). Each material was seeded with 50,000 cells and cultured with DMEM supplemented with 10% FBS and ascorbic acid; for mineralization assays, β-glycerophosphate (5 mM) was also added. Materials were tested at 1, 7, and 14 days of cell culture. For mineralization assays, materials were tested at 28 days of cell culture. 

### 2.4. Osteoblast Viability and Proliferation

The MTS assay was used to measure cell viability and proliferation by using the CellTiter 96^®^ AQueous One Solution Cell Proliferation assay (Promega, Alcobendas, Spain), according to the manufacturer’s protocol. 50 μL of MTS were added in each sample cultured with 250 μL of supplemented medium and incubated for 3 h. A scanning multi-well spectrophotometer was used to measure the absorbance at 490 nm.

The LIVE/DEAD Viability/Cytotoxicity Assay Kit for Mammalian Cells (Invitrogen, Carlsbad, CA, USA) was performed to characterize cell viability, attachment, and distribution. The LIVE/DEAD assay was performed by adding 300 μL of a solution at 4 μM EthD-1 and 2 μM of calcein AM in PBS per sample. Materials were incubated for 30–45 min at room temperature. Then, cells were observed with an Olympus BX61 microscope. Micrographs were taken and processed with Fiji software. Live and dead cells were simultaneously stained with green fluorescent calcein-AM and red fluorescent ethidium homodimer-1, respectively. 

### 2.5. Osteoblast Mineralization Assessment

Materials were washed with PBS and fixed with 10% formalin for 10 min. After that, materials were washed once again with PBS and stained with 300 μL of 40 mM Alizarin Red solution, with a pH of 4.2 (Sigma-Aldrich, St. Louis, MO, USA), per well at room temperature for 10 min under gentle shaking. The unincorporated dye was removed, and materials were washed carefully with PBS to remove excess stain. Then, mineralization was quantified by dissolving the precipitated Alizarin red in a 10% cetylpyridinium chloride solution at room temperature for 30 min with gentle shaking. An Infinite M200 scanning multi-well spectrophotometer (Tecan, Männedorf, Switzerland) was used to quantify the light absorbance at 550 nm of 100 μL of the stained solutions.

### 2.6. Osteoblast Morphology

Phalloidin–Tetramethylrhodamine B isothiocyanate (Sigma-Aldrich, St. Louis, MO, USA) was used for staining the cell cytoskeleton. Cells seeded onto materials were washed twice with PBS and fixed for 10 min in a 3.7% formaldehyde (Probus, Cornwall, UK) solution in PBS. Next, cells were washed extensively in PBS and permeabilized with 0.1% TRITON^®^ X-100 (Sigma-Aldrich, St. Louis, MO, USA) in PBS for 5 min, and gently rinsed with PBS. After that, cells were stained with 50 mg/mL of fluorescent phalloidin and 4′,6-diamidino phenylindole (DAPI) (0.2 mg/mL) (Sigma-Aldrich, St. Louis, MO, USA) in PBS (protected from the light) for 40 min at room temperature. Cells were observed with the Olympus BX61 microscope, and the micrographs were processed with Fiji software [26].

### 2.7. Bacterial Culture Preparation

To evaluate the antibacterial activity of the biomaterials, bacterial suspensions of *S. aureus* (CECT239) and *E. coli* (CECT405) were used at an approximate concentration of 10^6^ CFUs/mL (colony-forming units). To obtain this bacterial concentration, several steps were performed: First, the bacterial cultures from colonies in agar plates stored at 4 °C or from aliquots in cryopreservation were incubated overnight in Luria Bertani (LB) culture medium (10 g NaCl, 10 g tryptone, 5 g yeast extract in 1 L of distilled water) in a shaker (INFORS HT) at 37 °C and 200 r.p.m. Later on, a subculture was carried out for 1.5–2 h. A spectrophotometer (Infinite M Nano, TECAN) was used to monitor the optical density (OD) until reaching an OD600nm ~0.4. Thereafter, a 1:100 dilution of the subculture in a final volume of 1 mL of fresh medium was placed in each well of a 48-well plate (with the biomaterial to be tested). Then, plates were incubated at 37 °C and 100 r.p.m. for 24 h and 48 h. Negative controls (CTRL−) were represented by materials without bacteria inoculation, while positive controls (CTRL+) consisted of bacterial suspensions without materials.

Both, OD measurement and CFU counting are two different ways to evaluate bacteria growth. OD reads the turbidity of the culture media which measures the total bacteria expanding during the culture time. Otherwise, CFU is a unit that estimates the number of bacteria in a sample that are viable (living bacteria) and therefore able to multiply at this time.

### 2.8. Antibacterial Effect by Ion Diffusion

In order to evaluate the inhibition of bacterial growth by diffusion of Cu ions in the LB medium, the OD 600 nm of bacterial suspensions at the end of the incubation for 24 h and 48 h were recorded. Moreover, CFU counting was performed by plating 10 µL of culture dilutions (10^−5^ and 10^−6^ for *S. aureus* and *E. coli*, respectively) on LB agar plates (LB culture medium plus 15 g/L agar) and incubating overnight at 37 °C. The colony count from each dish was performed manually or using the Image J software (version number 1.53 s). CFUs/mL were calculated using the following formula:

(1) $$\mathrm{CFU}=\frac{\mathrm{number}\;\mathrm{of}\;\mathrm{colonies}}{\mathrm{dilution}\;\mathrm{factor}\cdot \mathrm{volume}\;\mathrm{of}\;\mathrm{culture}\;\mathrm{plated}}$$

### 2.9. Inhibition of Biofilm Formation

To evaluate the effect of copper on biofilm formation, bacterial cultures in 48-well plates were set up as described in Section 2.6 to perform two types of assays: crystal violet (CV) and LIVE/DEAD assay. To perform the CV staining, after 24 h or 48 h of bacterial incubation, materials were moved to a new 48-well plate, washed twice with PBS, and incubated with 0.1% CV for 15 min at room temperature with gentle shaking. After the CV staining, the excess was removed from the materials by washing them twice in PBS. Subsequently, absolute ethanol was added to each well for 10 min in order to dissolve the CV. Finally, the absorbance was measured at 570 nm. 

The LIVE/DEAD assay was performed using the Filmtracer™ LIVE/DEAD™ Biofilm Viability Kit (Thermo Fisher Scientific, Waltham, MA, USA). At the end of incubation, materials were moved to a new 48-well plate, immersed in a solution containing SYTO-9/Propidium Iodide 1:1 (6 μL in 1 mL of distilled and filtered water), and incubated for 20 min at room temperature and in the dark by gentle shaking. Then, materials were washed with PBS, and micrographs were obtained using a Nikon Eclipse Ni-E microscope. Micrographs were analyzed with ImageJ for fluorescence quantification by separating the green and red channels and calculating the number of white pixels in each channel corresponding with the live (green) and dead (red) bacteria in the image. Background material detected in the negative control (CTRL−)—material without cells—was extracted from each biomaterial image. 

### 2.10. Statistical Analysis 

For osteoblast assays, statistical analyses were performed by Wilcoxon or Mann–Whitney tests in SPSS version 22.0 for Windows. All in vitro tests were carried out three times with independent cell lines to ensure reproducibility. Each set contained three replicas of each sample and was investigated together with positive and negative controls. The results were normalized by the reference data (L-PBF Ti6Al4V(ELI) condition) within each experiment and each time to reduce the inter-experiment variability. The normalization of the results allowed the comparison of values between different days and the repeatability of experiments where different primary cell lines were used in each experiment.

For bacteria assays, statistical analyses were performed using one-way ANOVA with Tukey’s test for intergroup comparisons using GraphPad Prism version 9.0. The ion diffusion data were analyzed by subtracting the OD value of the CTRL− and normalized with the CTRL+ average. All experiments were performed in triplicate, with each one being repeated at least three times. Differences with a *p*-value < 0.05 were considered statistically significant. 

## 3. Results

### 3.1. Microstructural Analyses

**Figure 1:**
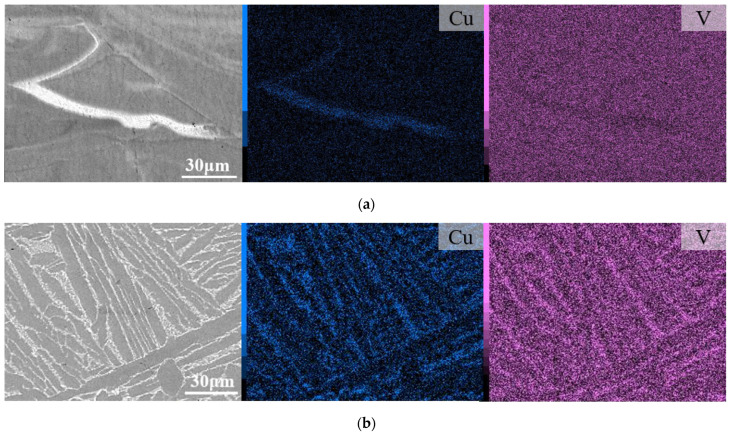
BSE-SEM micrographs after (**a**) SR and (**b**) HT of in situ-alloyed L-PBF Ti6Al4V(ELI)-3Cu, with their corresponding V and Cu EDX elemental maps.

SEM-EDX investigations of Ti6Al4V(ELI)-3Cu (SR) show that Cu was well dissolved in the matrix, although a few Cu-enriched areas were observed and associated with the melt pool boundary (Figure 1a). After HT, α′ transforms into α + β and V and Cu atoms were expulsed to α boundaries (Figure 1b).

**Figure 2:**
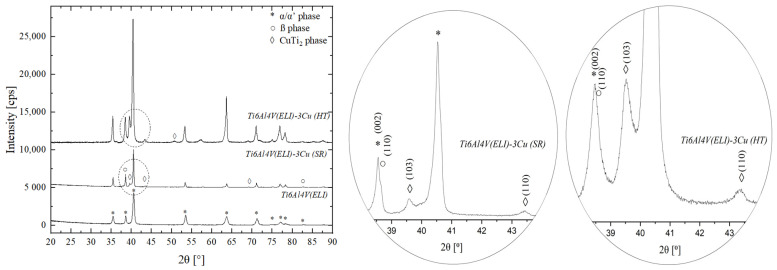
XRD pattern of L-PBF Ti6AL4V(ELI) and Ti6AL4V(ELI)-3Cu after SR and HT.

XRD measurements were performed to identify the phases of the in situ alloyed material after SR and HT. Most of the peaks were identified as the α/α′ phase, but the β and CuTi_2_ phases were identified after the addition of Cu (Figure 2). The peaks were identified in the International Centre for Diffraction Data (ICDD) with the following patterns: 00-044-1294, 01-089-3726, and 01-077-7746, respectively. The addition of Cu enhanced the formation of the β phase. Although the α/α′ phase (002) peak overlapped with the β phase (110) peak, an increase in peak intensity was observed after the addition of Cu at 2θ = 38.495°, due to the presence of the β phase. Therefore, the precipitation of the CuTi_2_ intermetallic phase was observed in SR materials, and it was enhanced after the performance of the HT. The unindexed peak of about 57.23° may belong to Cu_3_Ti_2_ phase, but, since other peaks of this phase were either not observed or overlapped with the peaks of other phases, a confident phase identification was not possible.

**Figure 3:**
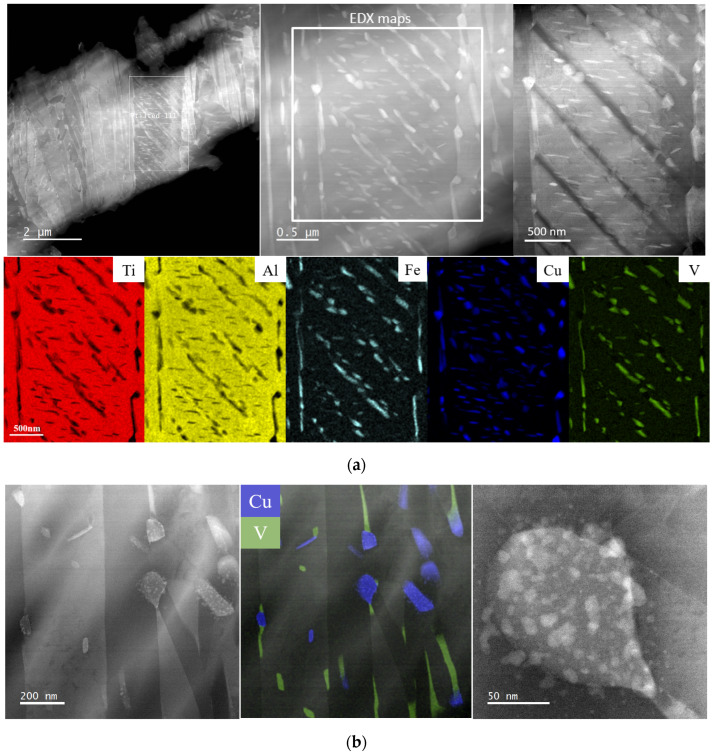
(**a**) STEM HAADF micrographs at low and higher magnification and the corresponding X-ray (EDX) elemental maps to highlight the beta and CuTi_2_ precipitates; (**b**) STEM HAADF (**left**) and composed image of V and Cu EDX maps (**middle**) as well as HAADF at high resolution (**right**) of a CuTi_2_ showing the progressive inhomogeneous segregation of Cu.

**Figure 4:**
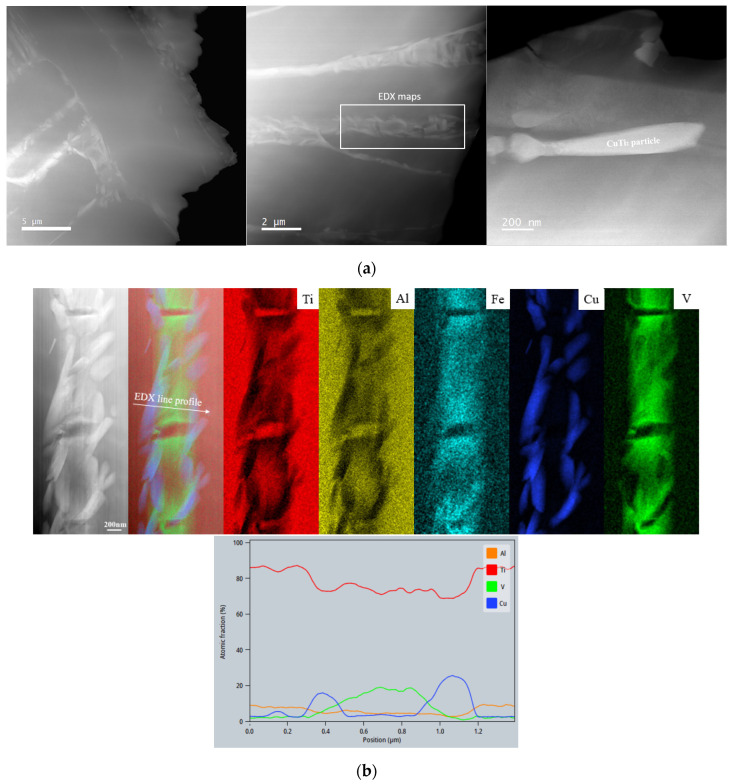
(**a**) STEM HAADF images of the HT samples showing the coarse microstructure (**left** and **middle**) and the coarse beta phase (**right**). Inset on the middle image depicts the region from which the EDX maps in (**b**) were required; (**b**) EDX maps and a line scan across the interlinked beta and CuTi_2_ phases.

General STEM observations of L-PBF Ti6Al4V(ELI)-3Cu after SR and HT are shown in Figure 3 and Figure 4, respectively, together with the EDX mapping analysis. After SR, thin α′ martensite laths (below 1.5 μm) were observed, as well as the precipitation of submicrometric precipitates located along α´ boundaries but also within them (Figure 3a). The elongated and spherical precipitates showed a homogeneous distribution within the matrix. The elongated precipitates enriched with V correspond to the β phase, whereas the spherical ones were identified as CuTi_2_ intermetallic precipitates [12]. They had a size of around 200 nm long and a diameter below 100 nm, respectively. Figure 3b shows high-magnification micrographs of a CuTi_2_ precipitate, whose morphology suggests that it was formed after the precipitation of even smaller Cu-enriched particles. β and CuTi_2_ intermetallic precipitates were mostly found interconnected with each other, although independent precipitates were spotted too. Such a connection between V and Cu elements can be associated with Cu as it is a β phase stabilizer.

The HT led to a change in microstructure (Figure 4). After heating up to the β region (above ~820 °C) [12], new α, CuTi_2_ intermetallic, and retained β were formed during the cooling. This resulted in α phase laths microstructure of a size above 4–5 μm, as well as Cu and V enriched precipitates between α laths (Figure 4a). Compared to the SR condition, β and CuTi_2_ intermetallic precipitates were completely interconnected after the HT (Figure 4b), and Cu precipitates changed to an elongated-shape morphology. β and CuTi_2_ intermetallic precipitates increased in size from an elongation of 200 nm to 1.5 μm and from a diameter below 100 nm to an elongated shape of a length of 1.2 μm, respectively. After the HT, precipitates were only visible between α laths. The EDX mapping confirms the precipitation of the CuTi_2_ intermetallic phase. The connection between β and CuTi_2_ phases is probably due to the diffusion process of Cu atoms, as Cu is a β stabilizer. The line profile in Figure 4b shows the element gradient across the β and adjacent CuTi_2_ phase. Since the CuTi_2_ phase in the micrograph is smaller than the thickness of the sample, it is difficult to extract the exact contribution from the Ti matrix volume for this particular area, and thus the elemental composition directly read from the graph does not exactly fit CuTi_2_. However, EDX spectra acquired from a thinner region show a Cu/Ti ratio of 2.1 (Ti: 65.94 at.% +/−7.36, Cu: 30.31 at.% +/−3.5, V: 1.5 at.% +/−0.38, Al: 1.76 at.% +/−0.36, Fe: 0.49 at.% +/−0.13).

Regarding the presence of Fe, it is already known that the Ti6Al4V alloy composition contains small traces (>0.1 wt.% in most Ti6Al4V alloys). The EDX elemental maps in Figure 3b and Figure 4b show the Fe to be evenly distributed in the β phase, thus acting as a β stabilizer. After HT, an increase in Fe concentration was observed due to Fe diffusion through dislocations (or other defects present in the matrix).

### 3.2. Osteoblast Assessment

#### 3.2.1. Evaluation of Cell Viability and Proliferation

**Figure 5:**
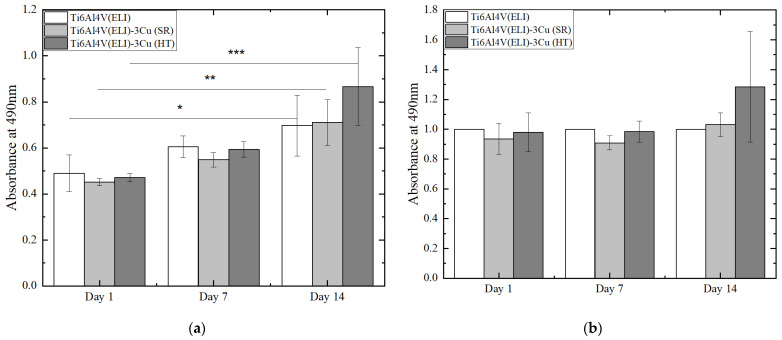
MTS assay of osteoblast cells at 1, 7, and 14 days of cell culture on the biomaterials. (**a**) Non-normalized and (**b**) normalized results by the Ti6Al4V(ELI) sample for each studied day. (n = 6; * *p*-values < 0.05, ** *p*-values < 0.01; *** *p*-values < 0.001).

Results of osteoblast viability and proliferation, assessed by MTS assay, are shown before and after normalization in Figure 5a,b, respectively. The non-normalized values allow for the analysis of cell proliferation by comparing the number of viable cells on each material among the different tested times, meanwhile normalized results are suitable for a better comparison of the number of viable cells among materials at each studied day. Figure 5a shows an increase in cell number across culturing time in all of the tested materials demonstrating that cells are able to proliferate on these surfaces. On the other hand, no statistically significant differences were observed between different materials at any time (Figure 5b).

**Figure 6:**
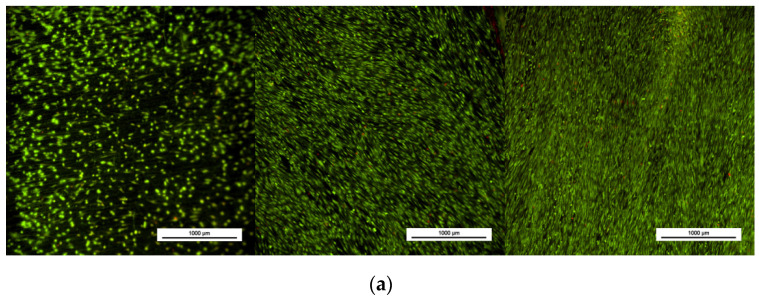

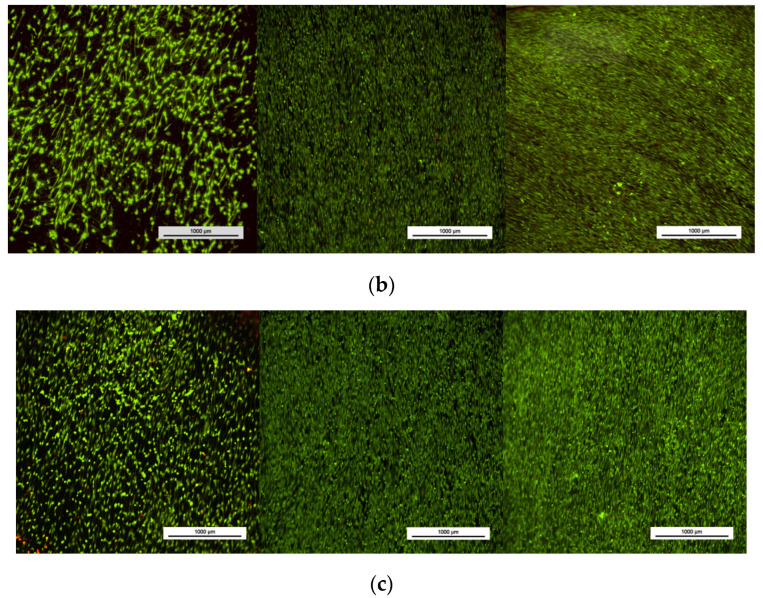
LIVE/DEAD assay at 1, 7, and 14 days of cell culture (from left to write) of (**a**) Ti6Al4V(ELI) and Ti6Al4V(ELI)-3Cu after (**b**) SR and (**c**) HT (n = 6).

In parallel, cell viability was assessed by fluorescent staining of live and dead cells. Figure 6 shows viable osteoblasts attached to the biomaterial surface in all cases. An increase in cell number is appreciated across time which matches with the MTS results. No significant number of dead cells was found in any of the materials at any of the measured times.

#### 3.2.2. Osteoblast Mineralization

**Figure 7:**
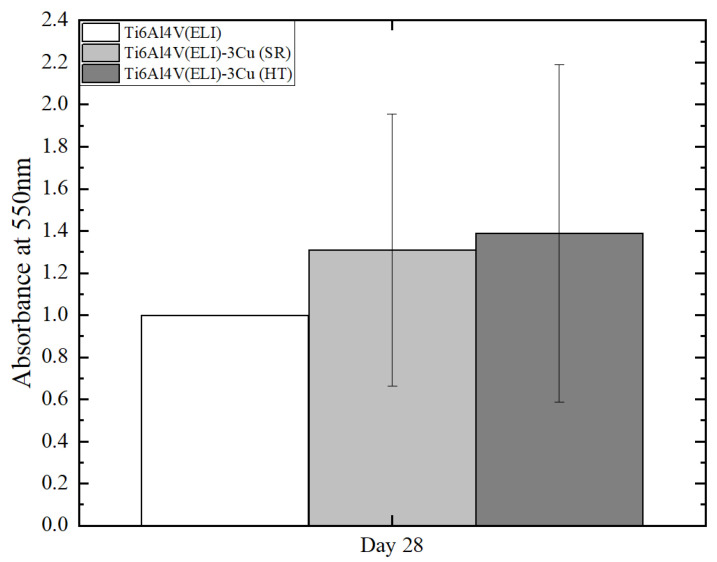
Alizarin red staining mineralization assay at 28 days of cells of Ti6Al4V(ELI) and Ti6Al4V(ELI)-3Cu after SR and HT; (n = 6).

After 28 days of culture, specimens were stained with Alizarin red in order to observe the mineralization capacity of osteoblasts to produce an extracellular matrix. The OD signal average appears to be higher in Cu-containing specimens. However, no statistically significant differences were found between the three conditions (Figure 7).

#### 3.2.3. Osteoblast Morphology

**Figure 8:**
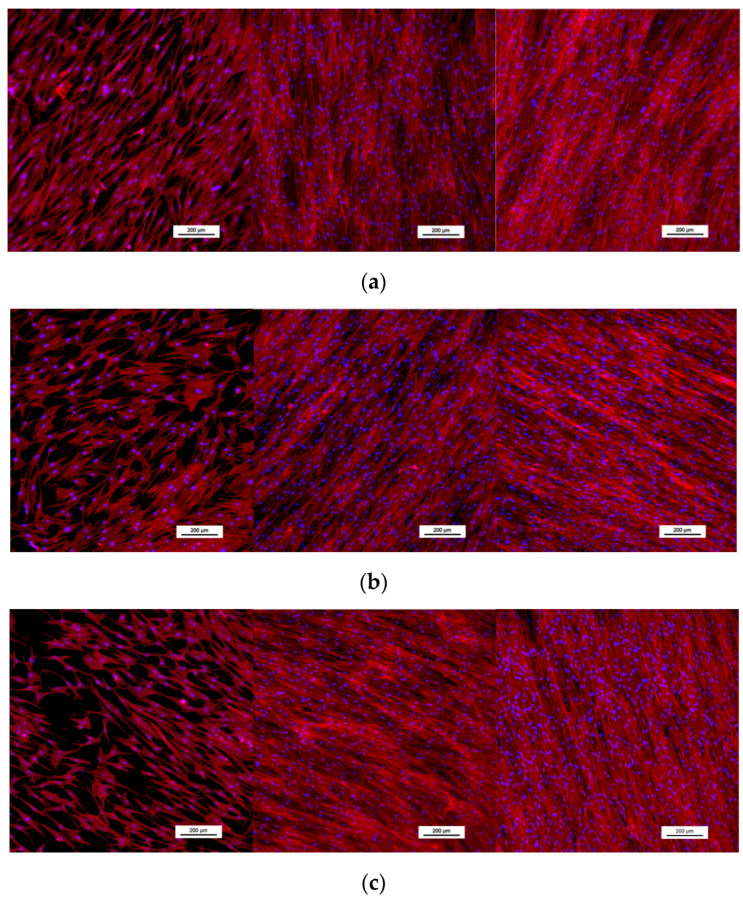
Staining of osteoblasts with Phalloidin (red) and DAPI (Blue) at 1, 7, and 14 days of cell culture (from left to right) of (**a**) Ti6Al4V(ELI) and Ti6Al4V(ELI)-3Cu after (**b**) SR and (**c**) HT.

Phalloidin staining was used to observe the morphology and placement of osteoblasts seeded on each material. Cells showed an elongated morphology and were aligned parallel to the ground lines present on the surface of the material, particularly in the later days of cell culture (Figure 8). At 14 days of culture, cells had packed themselves close together, covering the surface of the material. No differences among biomaterials were observed.

### 3.3. Bactericidal Assessment

#### 3.3.1. Evaluation of the Antibacterial Effect of Cu by Ion Diffusion

**Figure 9:**
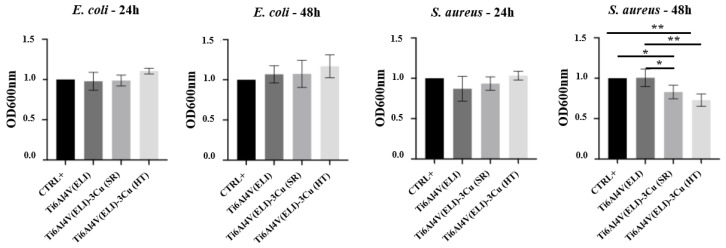
Representation of OD 600 nm average of the tested materials after 24 h and 48 h of *E. coli* and *S. aureus* incubation (n = 5; * *p* < 0.05; ** *p* < 0.01). Data of each biomaterial were normalized with the CTRL+ value.

Bacterial strains were cultured in the presence of materials for 24 or 48 h. OD assessment of suspension bacteria showed no significant differences for *E. coli* among the studied conditions. On the other side, *S. aureus* growth was significantly reduced after 48 h on materials containing Cu (Figure 9).

**Figure 10:**
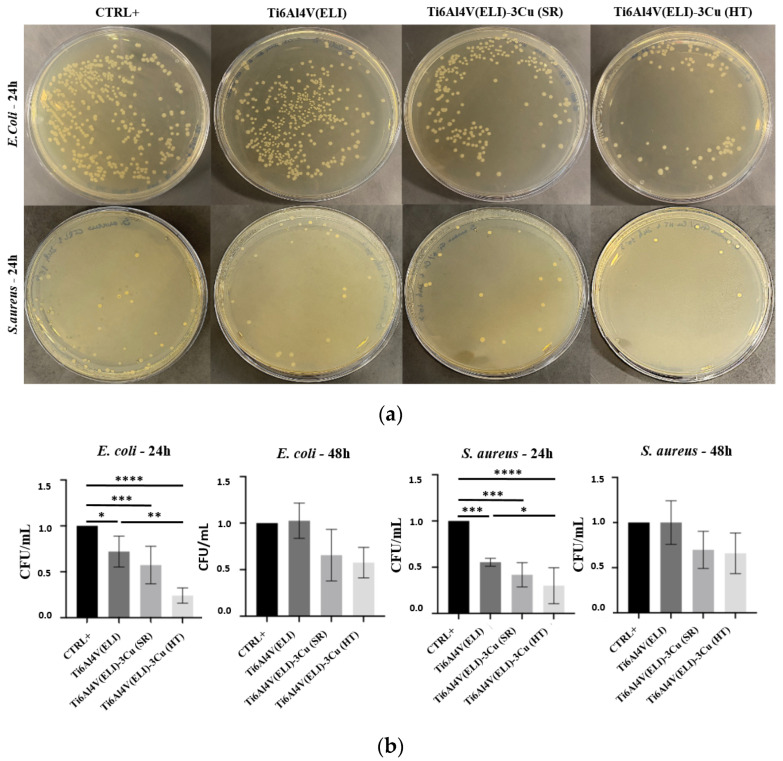
(**a**) Images of *E. coli* and *S. aureus* colonies after 24 h incubation; (**b**) normalized CFU/mL average variation of the studied materials after 24 h and 48 h (n = 5; * *p* < 0.05; ** *p* < 0.01; *** *p* < 0.001, and **** *p* < 0.0001). Data of each biomaterial were normalized with the CTRL+ value.

According to the quantification of CFU/mL, significant differences were observed at 24 h in the *E. coli* and *S. aureus* growth (Figure 10). For both strains and compared to CTRL+, Ti6Al4V(ELI) (material without Cu) already showed an inhibitory effect, probably due to the other alloying elements (Al and V). The inhibitory effect becomes stronger and more significant with the addition of Cu, and even more after the performance of the HT. No significant differences were found when comparing materials containing Cu (with and without HT). Among all the materials, Ti6Al4V(ELI)-3Cu (HT) showed the strongest inhibitory effect for *E. coli* and *S. aureus* after 24 h.

#### 3.3.2. Inhibition of Biofilm Formation

**Figure 11:**
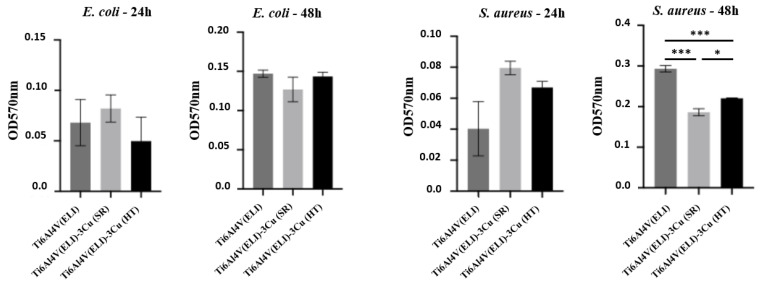
Optical density measurements of crystal violet, staining representing the amount of biofilm formed by *E. coli* and *S. aureus* at 24 h and 48 h on the surface of the studied materials. Error bars correspond to standard error. (n = 5; * *p* < 0.05; *** *p* < 0.001).

The formation of biofilm at 24 and 48 h of *E. coli* and *S. aureus* seeded onto biomaterials was evaluated by crystal violet assay (Figure 11). For *E. coli*, no significant differences were found among materials. On the other side, a significant reduction in *S. aureus* biofilm at 48 h was observed for materials containing Cu, mainly in Ti6Al4V(ELI)-3Cu (SR).

**Figure 12:**
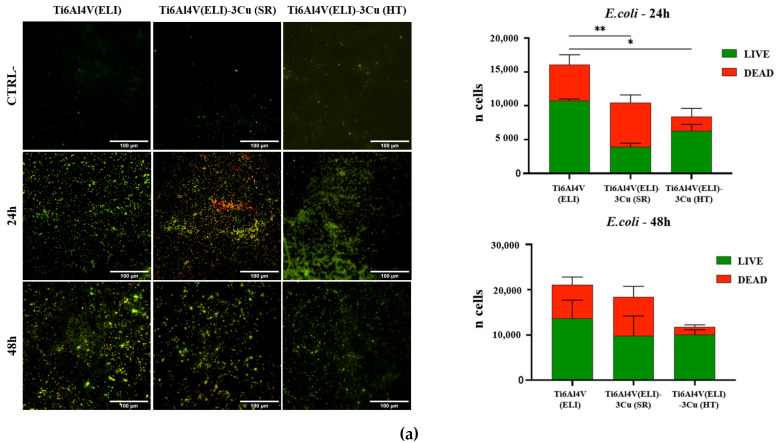

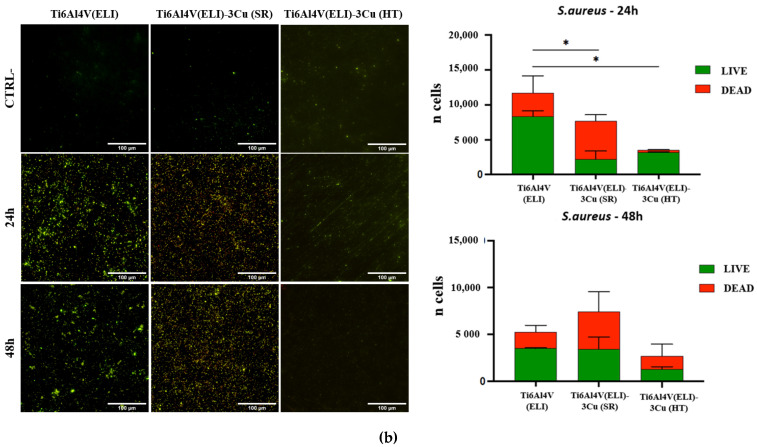
LIVE/DEAD qualitative micrographs and graphic representation of live and dead bacteria counts at 24h and 48h incubation of the studied specimens (n = 5; * *p* < 0.05; ** *p* < 0.01). (**a**) *E. coli*; (**b**) *S. aureus*.

The LIVE/DEAD results showed a significant reduction in living cells for both bacteria strains at 24 h in the presence of Cu with and without HT, while no significant differences were observed at 48 h, although a trend is observed in the HT biomaterial (Figure 12).

## 4. Discussion

In the field of orthopedic surgery, it is a promising strategy to use biomaterials with enhanced functionality to reduce the risk of failure of joint prostheses (e.g., by reducing the risk of infection). Cu is well known for its bactericidal activity [6,7]. Its effectiveness depends on factors such as: (i) the type of bacteria (Gram-negative or Gram-positive bacteria); (ii) experimental conditions; and (iii) amount and state of Cu content. In previous research, L-PBF Ti6AL4V(ELI) and Ti6Al4V(ELI)-3 at.% Cu were successfully manufactured and studied for their microstructure and mechanical properties [12,27]. Additionally, the effect of surface composition and roughness on osteoblast behavior was also evaluated [6]. The following discussion focuses on the effect of post-heat treatments on the microstructure as well as on the biological response.

### 4.1. Microstructural Analysis

The SR and HT post-treatments were carried out to relieve stresses and initiate phase transformations in the material. For Ti6Al4V, martensitic transformation is achieved at rapid cooling (e.g., as-printed Ti6Al4V), whereas α phase is an equilibrium phase formed after HT or SR and slow cooling. Both, α and α′ have the same hcp structure. When comparing L-PBF Ti6Al4V(ELI) material with the alloy manufactured with an addition of 3 at.% Cu, both after SR treatment, some differences in microstructure can be concluded. The addition of Cu leads to: (i) thicker needles of the α′ martensitic phase, (ii) a slightly higher amount of the β phase, (iii) the appearance of few rounded α grains within a size range of about 10 μm, and (iv) the formation of CuTi_2_ intermetallic precipitates [12,19]. The amount and ratio between phases in Ti–Cu alloys are important since changes in the Cu distribution and Cu ion release affect the antibacterial property of the alloy [13].

The SR treatment was carried out at a low temperature (at 650 °C for 3 h). Under equilibrium conditions, α + β microstructure should be obtained after a slow cooling from the β region (above ~820 °C) [12]. However, the initial microstructure of the material was dominated by the α′ martensitic phase, β phase, and CuTi_2_ phase. The heating up of the initial microstructure to α + β region resulted in some decomposition of α′ martensitic phase and the formation of some more Fe-rich, β, and CuTi_2_ phases. These CuTi_2_ intermetallic precipitates were formed between lamellas, but also within them (maybe on defects like twins or dislocations). Thus, a full homogeneity of the material was not observed. The formation of small amounts of secondary phases was correlated to the equilibrium phase diagram [12]. 

The HT was carried out at a high temperature (at 950 °C for 2 h). At 950 °C, the material has a β structure with a relatively homogeneous distribution of all elements [12]. The homogenization of the elements takes place during dwell time, and the formation of the final microstructure occurs during slow cooling, leading to the formation of coarse lamellae α phase. These lamellae are coarser than the ones formed during SR treatment. This fact can be explained by the higher rate of diffusional processes at higher temperatures. Therefore, due to the lower solubility of V, Fe, and Cu in α, those elements diffuse to α boundaries forming Fe-rich, β and CuTi_2_ phases. An increase in β as well as in CuTi_2_ intermetallic precipitates fraction and size were observed after the HT compared to SR treatment. Additionally, higher Fe concentrations after HT compared to SR are due to its diffusion through dislocations or other defects present in the matrix. For L-PBF Ti6Al4V(ELI) without Cu, it has been reported that the performance of an annealing treatment at 950 °C for 2 h leads to the decomposition of α′→ α + β. Martensitic α′ phase transforms into α phase, displacing V atoms to α boundaries [28]. In addition, a lower amount of α phase but coarser α lamellas have been obtained within the same study at higher temperature HT but below β transus. For Ti6Al4V, it has been reported that the β fraction at high temperatures is larger, reducing the equilibrium α fraction from approximately 87% at 780 °C to 73% at 850 °C and 23% at 950 °C [28]. 

### 4.2. Osteoblast Behavior 

Generally, the best biological performance of Ti-based Cu alloys has been found for small amounts of Cu content, where bactericidal behavior is exhibited without compromising cell integrity. In vitro studies on Ti-10 wt.% Cu material have been performed with MC3T3 and *Staphylococcus epidermidis*. Their sensitivity was found to be between 9 × 10^−5^ g/mL and 9 × 10^−6^ g/mL of Cu ions in solution. The optimal growth of bacteria occurred in the range of 9 × 10^−6^ g/mL to 9 × 10^−7^ g/mL, while low survival of MC3T3 was found at Cu concentrations above 9 × 10^−5^ g/mL [29]. 

The cytocompatibility of Ti6Al4V-5 and 6 wt.% Cu has been reported to be equivalent to the Ti6Al4V alloy, which is generally recognized as a safe material and is already approved for biomedical applications [13,30]. Ti6Al4V-6 wt.% Cu alloy did not negatively affect the cell viability of gingival fibroblasts and osteoblasts. It inhibited the inflammatory response of macrophages, increased the angiogenesis properties of HUVECs, and decreased local inflammatory responses by inhibiting macrophages [31]. In the present study, for 3 at.% Cu, osteoblasts proliferated comparably between days 1 and 14 for all materials. Although no statistically significant difference in cell proliferation was found between materials, the higher OD average on Ti6Al4V(ELI)-3 Cu (HT) after 14 days of cell culture should be noted. This is probably due to the osteogenic properties of Cu since Cu ions release easier from β than α phase [13]. The same results were observed in the mineralization capacity of osteoblasts without differences among materials but with a slight increase in 3 at.% Cu materials. In a previous study using the CoCrMo alloy, it was found that the addition of 2 wt.% Cu could induce osteoblast proliferation and differentiation as well as inhibit osteoblast apoptosis [31].

The study of cell morphology showed good attachment in all materials, with a flattened and elongated cytoskeleton, no discernible differences between materials, and cell packing and confluence at later dates of culture. These results were as we expected, as the Cu concentration in the alloyed material is considerably lower than previously reported cytotoxic amounts [21].

### 4.3. Bactericidal Behavior 

In our study, the bactericidal behavior of Cu and HT was evaluated using different approaches: inhibition of bacterial growth in suspension and biofilm formation. In the first case, the release rate of Cu ions to the media is a key factor, while in the other case, the surface microstructure confers the ability of Ti6Al4V(ELI)–Cu to inhibit biofilm formation at early stages. Antimicrobial agents can act and affect differently upon planktonic or biofilm organisms; therefore, susceptibility must be determined preferably under conditions that simulate conditions in vivo. For example, biofilm-associated organisms grow more slowly than planktonic organisms, probably because the cells are limited by nutrient and/or environmental anoxia. Interestingly, within the biofilm, the bacteria adapt to this environment by altering their metabolism, gene expression, and protein production. In addition, these adaptations provide the bacteria with resistance to antimicrobial agents by inactivating the antimicrobial targets or reducing the accessibility to the immune system. Hence, the main challenge of a biomaterial is to avoid biofilm formation at the first stages. The main results of this work showed that planktonic growing according to the CFU data obtained from 24 h of culture, Ti6AL4V(ELI)-3 at.% Cu (HT) biomaterials seemed to have an inhibitory effect compared to control and Ti6AL4V(ELI) without Cu both in *E. coli* and *S. aureus*. Compared to Ti6Al4V(ELI) alloy, the addition of 3 at.% Cu does not show a significant bactericidal effect after SR treatment. These results can be correlated with differences in microstructure, as Cu ions have been reported to be easier to release when they are in solid solution in the β phase state compared to α solid solution or the CuTi_2_ intermetallic phase [13,18,20]. Results obtained by crystal violet staining suggest a significant reduction in biofilm formation in both Ti6Al4V (ELI)-3 at.% Cu compared to Ti6AL4V(ELI) without Cu at 48 h only in *S. aureus*. According to LIVE/DEAD assay on biofilm formation, the *E. coli* and *S. aureus* viability was affected in both Ti6Al4V (ELI)-3 at.% Cu. In situ alloying of Ti6Al4V–Cu via L-PBF showed that the addition of low percentages of Cu (1–6%) decreased bacterial growth without causing cell cytotoxicity [32]. The results of this study showed that Ti6Al4V(ELI) without Cu already provides a decrease in bacteria compared to CTRL+, probably due to the release of V ions [33]. 

The microstructure observed after SR and HT treatments is very different, mostly in terms of the size and distribution of β and CuTi_2_ precipitates. SR is composed by α/α′ matrix with homogeneously distributed small β and CuTi_2_. The performance of HT leads to the redistribution of β and CuTi_2_ phases along α boundaries, as well as an increase in the size of β and CuTi_2_. Peng et al. [20] reported that most likely larger CuTi_2_ or β phase precipitates are more efficient at inhibiting the growth of bacteria that are adhered to the surface of the alloys. In that study, two HTs were performed, the first one at 740 °C and the second one at 910 °C, both followed by air cooling. The first HT showed a microstructure composed of globule CuTi_2_ precipitates and β phase with sizes of 400–700 and 1000–2000 nm, respectively, while the second one showed an average size of 50–80 nm and 100–200 nm, correspondingly. Results showed that the antibacterial performance of larger globule CuTi_2_ precipitates and the β phase was better than the small ones since the size of the precipitates was closer to the size of *S. aureus* (500–800 nm). In the present study, significant bactericidal behavior was observed for both conditions with Cu (SR and HT conditions) compared to Ti6Al4V(ELI) without Cu. No significant differences were observed between both post-heat treatments. Although the size of β or CuTi_2_ phases enhances the direct-contact killing mechanism, results suggest that the distribution of such phases into the matrix should be considered an important bactericidal factor. The size of *S. aureus* has been reported to be between 500 and 800 nm [20], but the minimum width found of α lamella after HT is about 4 μm, indicating that bacteria deposited within them will not be affected as the ones deposited on β or CuTi_2_ phases. Observations show that smaller but homogeneously distributed β or CuTi_2_ phases can be as effective as larger β or CuTi_2_ phases precipitates located along α′ boundaries. For Ti6Al4V-5.5 wt.% Cu, it has been reported that it suppressed biofilm formation, virulence, and antibiotic resistance of *S. aureus* [17]. Such behavior is attributed to the ability to inhibit biofilm formation at early stages. This is an important finding, as biofilms are more resistant to antibiotics and the body’s immune response than planktonic bacteria. In this context, Cu-containing biomaterials hold great promise in the fight against bacterial infections related to prosthetic implants. However, in-depth studies are required to explain the bactericidal mechanism and, to some extent, the interaction between the bacteria and the surface of the alloy. 

## 5. Conclusions

The microstructural features of L-PBF Ti6Al4V(ELI)-3Cu after SR and HT were studied, as well as their osteoblastic and bactericidal performances. The L-PBF Ti6Al4V(ELI)-3Cu (SR) materials had a microstructure with a small β phase and CuTi_2_ intermetallic precipitates of sizes below 100 nm and 200 nm, respectively. Both phases were homogeneously distributed with in α′ matrix. The performance of an HT at 950 °C for 2 h led to an increase in the amount and size of β and CuTi_2_ phases up to 1.2 μm and 1.5 μm, correspondingly.

The addition of 3 at.% Cu did not negatively affect the osteoblast performance. Additionally, it hinders the attachment of *S. aureus* and *E. coli* and reduces the formation of biofilm. No significant differences between SR and HT conditions were found. The homogeneously distributed β or CuTi_2_ precipitates within the α′ matrix after the SR treatment, compared to their localized distribution along α needles after HT, suggests that the distribution of β and/or CuTi_2_ phases within the matrix is important for the direct contact killing of *S. aureus* and *E. coli*. However, compared to Ti6Al4V(ELI), the addition of 3 at.% Cu led to significant bactericidal behavior by means of ion release after the performance of the HT. Results suggested that the increase in β and CuTi_2_ facilitate the release of Cu ions, showing bactericidal behavior against *S. aureus*, but especially against *E. coli*, after 24 h. 

Deeper studies on HT of L-PBF Ti6Al4V(ELI)–Cu alloys are highly suggested to completely understand the different possible microstructures of such alloys as well as their mechanical, corrosion resistance, and biological properties.

## Data Availability

Generated data during the study is available under request.
